# The potential role and research progress of the gut microbiota–NGF axis in postmenopausal bone homeostasis imbalance

**DOI:** 10.3389/fimmu.2026.1933248

**Published:** 2026-09-16

**Authors:** Yangyang Chen, Dingpeng Li, Zhong Wang, Jiawen Li, Haoze Zhang, Xingwen Xie

**Affiliations:** 1 Gansu University of Traditional Chinese Medicine, Lanzhou, China; 2 The Second People’s Hospital of Gansu Province, Lanzhou, China; 3 Affiliated Hospital of Gansu University of Traditional Chinese Medicine, Lanzhou, China

**Keywords:** bone homeostasis, gut microbiota-NGF-bone axis, gut microbiota, nerve growth factor, neuroimmune regulation, osteoporosis

## Abstract

Postmenopausal osteoporosis(PMOP)is a common metabolic bone disease characterized by an imbalance in bone remodeling due to estrogen deficiency. Increasing evidence suggests that alterations in gut microbiota, intestinal barrier, immune response, microbial metabolites, and autonomic nervous activity are involved in postmenopausal bone loss. Nerve growth factor (NGF) and its receptors tropomyosin receptor kinase A (TrkA) and p75 neurotrophin receptor (p75 NTR) are implicated in sensory nerve innervation, inflammation regulation, bone adaptation, bone repair, and pain sensitization, thus potentially serving as a molecular interface linking microbiota, nerves, immunity, and skeletal processes. This review evaluates the proposed gut microbiota–NGF–bone relationship by distinguishing between mechanisms supported by experimental evidence, indirect evidence, and speculative hypotheses. Existing studies support the regulation of NGF by gut microbiota under specific neuroimmune contexts, the gut microbiota–immune–bone interactions in estrogen deficiency models, and the roles of NGF and its receptors in neurobiology and skeletal biology. However, these findings originate from independent experimental systems. Currently, no studies have simultaneously demonstrated that gut microbiota-induced changes in NGF signaling are a necessary or sufficient condition for the abnormal bone remodeling associated with PMOP. Therefore, the gut microbiota–NGF–bone axis should be viewed as a conceptual framework connecting the microbiome, immunity, neuroendocrine factors, and bone metabolism, rather than as an established causal pathway. This framework aids in integrating existing evidence, clarifying mechanistic gaps, and proposing testable scientific questions. Future research should combine the manipulation of gut microbiota with tissue or cell-specific regulation of NGF, TrkA, or p75 NTR, while also conducting rescue experiments, longitudinal multi-omics analyses, and systematic skeletal phenotype evaluations. Direct causal validation must be obtained before applying gut microbiota or NGF-targeting strategies for the precise prevention and treatment of PMOP.

## 1 Introduction

Postmenopausal osteoporosis (PMOP) is a condition characterized by an imbalance in bone metabolism due to a sudden decline in estrogen levels, resulting in bone mass reduction and deterioration of bone microstructure (1). Epidemiological studies indicate that the prevalence of this condition among women over the age of 50 in China reaches 32.1%, exceeding 50% in those over 65. As the aging population in China intensifies, the incidence of fragility fractures due to osteoporosis has significantly increased, leading to a rising rate of disability and mortality, thereby imposing a heavy burden on families and society (2). It has been thought that primarily through endocrine-immunological pathways, estrogen deficiency affects bone homeostasis. On the one hand, the reduction of estrogen levels would act directly to weaken the function of osteoblasts. On the other hand, low-grade chronic inflammation comes up due to estrogen deficiency. This low-grade chronic inflammation will upregulate the pro-inflammatory factors such as tumor necrosis factor-alpha (TNF-α), interleukin-6 (IL-6) etc. They accelerate the differentiation and activation of osteoclasts (3, 4). In the last couple of years, the concept of the gut-bone axis has emerged to shed light on the pathogenesis of PMOP. Research has revealed that estrogen deficiency related to menopause can lead to dysbiosis of the gut microbiota, which, along with a decline in gut barrier function and translocation of endotoxins, activates the immune system and enhances osteoclastic signaling, ultimately exacerbating bone loss (5, 6). Concurrently, increasing evidence suggests that the gut microbiota interacts with the host’s nervous system through its metabolites and neuro-endocrine pathways, forming an important network that jointly regulates bone metabolism, referred to as the ‘gut microbiota -neuro-bone axis’ (7–9).

NGF is the first neurotrophic factor discovered and is situated at the intersection of neural development, inflammatory response, bone remodeling, and pain sensitization within the neural-bone regulatory network. Consequently, it may serve as an important candidate molecule linking gut microbiota dysbiosis with bone homeostasis imbalance (10, 11). Rich sensory and sympathetic nerve fibers are distributed in the periosteum, bone marrow, and trabecular bone, directly or indirectly regulating bone homeostasis through the release of neurotransmitters and neurotrophic factors such as NGF (12). NGF plays a crucial role in the development of peripheral sensory nerves and pain transmission. Furthermore, within the bone, NGF and its receptors TrkA and p75 NTR are also present on osteoblasts, osteoprogenitors, and osteoclasts, suggesting that NGF can exert its effects in the bone metabolic microenvironment through autocrine and paracrine mechanisms (13). Current research indicates that the overexpression of NGF promotes bone pain sensitivity and abnormal neural remodeling, with a focus on conditions such as osteoarthritis and bone metastatic pain (14, 15). Shang et al. found that under postmenopausal low estrogen conditions, the expression and release of NGF may undergo adaptive changes (16). Accordingly, existing evidence separately supports the “gut microbiota-immune-bone” pathway and the “NGF/receptor-nerve-bone” pathway; nevertheless, it remains unproven that NGF mediates the skeletal impacts of gut dysbiosis in PMOP. This review aims to systematically summarize the validated roles of gut microbiota-related immune and metabolic pathways in bone homeostasis, and evaluate the context-dependent functions of NGF in neuro-skeletal biology. In addition, it assesses the limitations of current gut microbiota-targeted interventions and puts forward corresponding experimental strategies.

## 2 Potential mechanisms by which gut microbiota may influence NGF-related signaling

### 2.1 Metabolic pathways

The metabolites formed by gut microbiota act as important mediators of communication with the host. Among these, short-chain fatty acids (SCFAs) are considered to be extremely important, as they regulate intestinal as well as immune functions and also influence the central nervous system through epigenetic and receptor pathways (17). According to Barichello et al., butyrate supplementation improves the expression of Brain-Derived Neurotrophic Factor(BDNF) in the hippocampus, can improve neurogenesis, and is beneficial for memory (18). The development of the neurotransmitter system may be mediated by several mechanisms: firstly, SCFAs have the potential to function as inhibitors of histone deacetylases thereby altering the epigenetic state of genes involved in NGF (17, 19); secondly, SCFAs may also affect the functioning of neurons and glial cells, through signaling of G-protein-coupled receptors such as GPR41/43 and indirectly inducing the secretion and production of NGF (20, 21). A gut microbiota that is healthy and diverse produces metabolites including SCFAs that may act to maintain levels of NGF and neurotrophic environment. However, imbalance in the microbiome can lead to a change in the metabolite profile. It is essential to understand that evidence regarding regulation of NGF by SCFAs is presently limited. Evidence mainly refers to SCFAs indirect effects on the expression of neurotrophic-related genes and glial cells’ functionality. Thus, the experimental validations of the aforementioned SCFAs, NGF, and bone homeostasis chain are to be performed.

### 2.2 Immune pathways

The gut microbiota can modulate NGF production through immune system intermediates. The colonization of gut microbiota during the neonatal and juvenile stages is particularly important for neuro-immune development. Research by Abdullah et al. indicates that early gut microbiota colonization is known to upregulate the production of NGF by mast cells, thereby sensitizing sensory neurons (22). Germ-free mice, lacking microbiota, exhibit reduced NGF expression and diminished pain sensitivity, and this relevant phenotype can only be restored by normal microbiota colonization at birth, with weak effects observed post-weaning. This phenomenon suggests a critical window for the interaction between microbiota, immunity, and neurons during early life, which is of great importance for NGF-mediated sensory nerve development. The specific mechanism involves the gut microbiota promoting the maturation of mast cells and the release of NGF, which subsequently binds to the TrkA receptors on nerve endings, facilitating the translocation of Transient receptor potential vanilloid 1(TRPV1) channels and thereby increasing neuronal sensitivity to harmful stimuli. This research also shows how NGF modulation by microbiota through immune cells impacts neural development in the long run (22, 23). In adult organisms, immune cells such as macrophages can also produce NGF, and this production is induced by microbe-associated molecular patterns (MAMPs) (24).

*In vitro* experiments have shown that human monocytes/macrophages can secrete NGF even in the absence of stimulation, and the synthesis of NGF further increases following stimulation with lipopolysaccharide (LPS). LPS, an endotoxin from Gram-negative bacteria, activates the nuclear factor kappa-B (NF-κB) and Mitogen-activated protein kinase(MAPK) pathways in macrophages through the TLR4 receptor, inducing the release of various inflammatory mediators, including NGF (25). When the intestinal barrier function is compromised, bacterial endotoxins enter the bloodstream, creating a systemic inflammatory environment, which may lead to an increase in NGF production by the monocyte/macrophage system (26). Reports of this phenomenon have also been observed in chronic inflammation and infection states such as rheumatoid arthritis, suggesting that NGF is associated with bone tissue repair and increased pain sensitivity (27, 28). These findings propose a hypothesis: the changes in NGF expression by immune cells induced by dysbiosis may further alter the bone marrow microenvironment under the background of chronic low-grade inflammation in postmenopausal women, thereby affecting the balance between osteoclasts and osteoblasts.

### 2.3 Neural pathways

The gut microbiota not only influences the nervous system through metabolites and immune mediators but also directly regulates the host’s nervous system via neural pathways, thereby indirectly involving NGF regulation (29, 30). Research related to the “gut-brain axis” shows that the gut microbiota can affect the activity of the vagus nerve, as well as the levels of neurotransmitters and neurotrophic factors in the central nervous system (31). After the oral administration of certain probiotics or pathogens, there is a direct stimulation of enteric neurons and sensory nerve endings in the intestinal mucosa, triggering vagal nerve discharge and central feedback (31, 32). Through this neural reflex pathway, microbial signals can carry out rapid actions on brain and peripheral organ function without the involvement of immune reactions (33). A study by Song et al. found that oral administration of specific bacteria to mice could activate the vagal nerve nucleus, increase the levels of NGF and BDNF in the brain, and improve their behavioral performance (34). Although much of the focus of these studies has been on central nervous system diseases, such as depression and cognitive dysfunction, their mechanisms suggest that the gut microbiota may influence the demand and utilization of factors like NGF in tissues throughout the body, including the skeleton, by altering autonomic nervous system activity.

The sympathetic and parasympathetic nerves in the autonomic nervous system play a regulatory role in bone metabolism, while the gut microbiota can modulate the tone of the sympathetic/vagal nerves, which is considered one of the pathways through which the gut microbiota influences the function of multiple host organs (35–37). Research by Qiao et al. found that certain changes in the composition of the gut microbiota are genetically associated with a decrease in bone density, partially mediated through heart rate variability (an indicator of autonomic nervous function) (38). This suggests that the impact of the gut microbiota on bone tissue may involve neural regulatory mechanisms. Thus, autonomic nervous regulation may be an important intermediary pathway by which gut microbiota affect bone homeostasis, at least with respect to NGF at the neuro-bone interface, but evidence for this pathway is currently scarce.

Overall, existing evidence suggests that the gut microbiota may regulate host NGF-related signaling through multiple potential pathways, including metabolic products, immune cells, and neural reflexes. However, most of the aforementioned pathways have been studied in largely independent experimental systems, and it has not yet been confirmed that the microbiota can mediate regulatory effects on the bone-neural network through NGF.

## 3 Regulation of NGF in postmenopausal bone homeostasis

### 3.1 Sources of NGF and receptor distribution in bone tissue

Bone is richly innervated by sensory and sympathetic nerve fibers, which not only release traditional neurotransmitters such as norepinephrine and substance P, but also neurotrophic factors like NGF to influence the function of bone cells (39). NGF itself is made by several different cell types, such as osteoblasts, fibroblasts, and inflammatory cells. Research indicates that mature osteoblasts co-express neurotrophin NGF and its high-affinity receptor TrkA suggesting the presence of a role for osteoblasts’ NGF (11). In addition, the low-affinity receptor p75 NTR (also recognized as NGFR) is further believed to be expressed in greater levels in bone cells (40). Compared to the low expression of TrkA in bone tissue, p75 NTR protein is more frequently present and abundant in osteogenic cells (41). Notably, p75 NTR expression is low in early-stage bone marrow mesenchymal stem cells, while its levels significantly increase in osteoblasts after induction of osteogenic differentiation (42, 43). This means that p75 NTR is likely the primary mediator of NGF signaling in bone, especially during the differentiation of osteoblasts/chondrocytes to mature forms. NGF receptors in bone tissue show a cell-specific expression distribution: TrkA is mainly on intraosseous nerve fibers and sensory neurons, whilst p75 NTR is relied on more by bone cells and mesenchymal progenitor cells to sense NGF.

### 3.2 Regulation of osteoblast differentiation and bone formation by NGF

As a growth-promoting factor, NGF under physiological or weak inflammatory conditions mainly promotes osteoblast differentiation, sensory neuron-osteocyte coupling, and the repair process in bone via TrkA-associated signaling (44, 45). According to a study by Zha et al., the enhancement of exogenous NGF can boost differentiation of Bone marrow mesenchymal stem cells (MSCs) into osteoblasts by activation of the TrkA receptor and downstream ERK/AKT signaling plasticity, upregulation of the osteogenetic genes as well as improving bone formation ability directly (46). In a mouse bone stress model, mechanical stimulation significantly upregulates NGF expression in osteoblasts on the bone surface within one hour, subsequently activating the TrkA receptors on adjacent sensory nerve endings, which prompts sensory nerves to release osteogenic signaling molecules (47). In mice with suppressed TrkA signaling, the new bone formation induced by mechanical loading is significantly diminished, accompanied by a decrease in Wnt/β-catenin activity within osteocytes. Conversely, administering additional NGF to normal mice further enhances bone formation and Wnt signaling triggered by mechanical loading (48, 49). Together, these findings support a proposed osteoblast–sensory nerve feedback model in mechanical-loading experiments: osteoblast-derived NGF activates sensory nerves, while nerve-derived signals, potentially including calcitonin gene-related peptide (CGRP), may enhance osteogenic pathways such as Wnt/β-catenin.

Furthermore, under physiological conditions, NGF may indirectly promote osteogenesis through vascular-neural coupling. The process of bone formation is often accompanied by angiogenesis and neural remodeling. Li et al. observed in a fracture model that after a fracture, nerve regeneration within the callus occurs earlier than angiogenesis, with both spatial trajectories highly coinciding. A peak in NGF expression was detected precisely between the peaks of neural and vascular growth (50). Based on this, it is speculated that NGF serves as a coupling signal linking neural and vascular regeneration, enabling nerves to guide vascular growth, thereby providing the necessary support for osteogenesis. During the processes of bone repair and remodeling, NGF not only directly promotes the activity of osteoblasts but also supports new bone formation indirectly by recruiting blood vessels and attracting progenitor cells.

### 3.3 Regulation of osteoclast differentiation and bone resorption by NGF

NGF does not always exhibit a bone-promoting effect in the regulation of bone homeostasis; rather, it can enhance osteoclast-related signaling under specific conditions, thus participating in the regulation of the dynamic balance of bone remodeling. Existing studies suggest that NGF can indirectly promote osteoclastogenesis by affecting the paracrine function of osteoblasts and bone marrow stromal cells. NGF stimulation specifically induces the expression of receptor activator of nuclear factor-κB ligand (RANKL), and downregulates osteoprotegerin (OPG) levels, which subsequently increases the RANKL/OPG ratio, enhancing osteoblast-mediated signaling for osteoclast precursor differentiation (50, 51). The impact could be significantly stronger in a situation where the inflammation is increasing (as may be the case in cancer). Indeed, the effect of pro-inflammatory factors and NGF signaling can have a synergistic effect on the RANKL/OPG ratio promoting osteoclast formation and bone resorption (52).

Besides the indirect regulation elaborated above, NGF may also directly act on osteoclasts and their precursors. Existing studies indicate that osteoclast precursor cells could express the TrkA receptor and binding with NGF could activate downstream signaling pathway such as NF-κB and MAPK that promote differentiation of these precursor cells into mature osteoclasts (53). In a similar manner, it is observed that in inflammatory context, pro-inflammatory mediator TNF-α up-regulates the surface expression of p75 NTR on osteoclast precursors thereby increasing their sensitivity to NGF stimulation. So that NGF will likely have an osteoclast-promoting effect in a pro-inflammatory setting (54, 55).

NGF’s regulation of bone metabolism is not defined by a fixed unidirectionality but rather shows high microenvironmental dependence. Under physiological or low-inflammatory conditions, NGF is more likely to undergo osteogenic differentiation, mechanical adaptation, and tissue repair; while under high-inflammatory conditions, altered receptor expression profiles and an imbalanced bone marrow microenvironment may lead to NGF’s involvement in osteoclastogenesis and bone resorption. In other terms, the functional consequence of NGF signaling depends not only on the type of receptor but also on the local level of inflammation and the dynamic balance of TrkA and p75 NTR. While no validated quantitative threshold currently defines the transition from osteogenic to osteoclastogenic NGF signaling, the proposed functional switch should be regarded as a context-dependent continuum rather than a binary mechanism. In light of this, NGF can be considered as an important regulatory node linking bone formation with bone resorption processes, whereby the inflammatory state is considered to be one of the determinants of the direction of action. **Figure 1** illustrates how NGF exerts a dissociative effect on bone metabolism depending on the microenvironments. As the inflammatory and receptor-expression context changes, the effective skeleton will shift from anabolic to catabolic.

**Figure 1:**
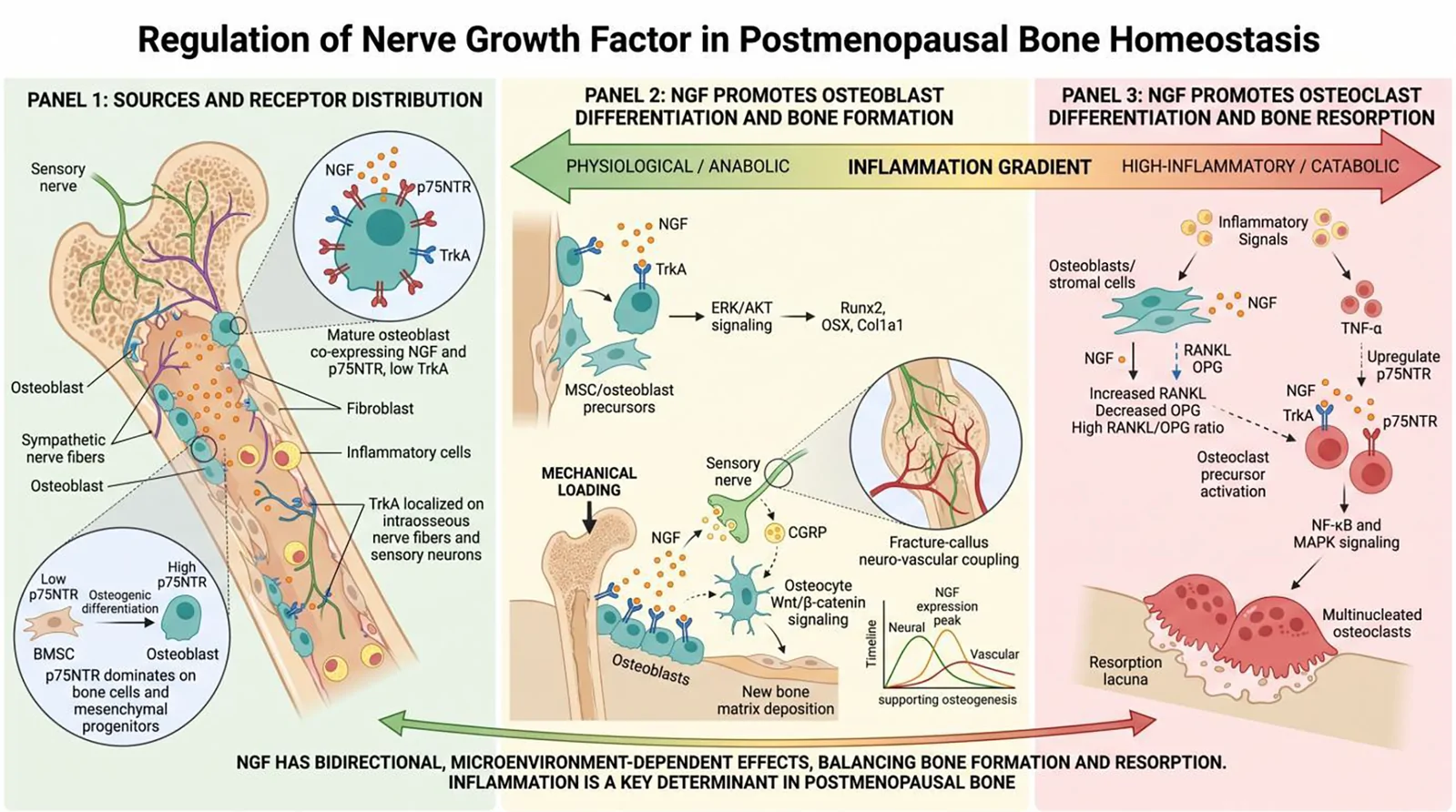
Context-dependent roles of NGF in postmenopausal bone homeostasis. NGF and its receptors TrkA and p75 NTR are distributed in skeletal, neural, stromal, and immune cells. Under physiological conditions, NGF/TrkA signaling supports osteoblast differentiation, neurovascular coupling, and bone formation; under inflammatory conditions, NGF-related signaling may promote osteoclastogenesis, bone resorption, and pain sensitization.

### 3.4 The impact of the postmenopausal microenvironment on NGF expression and function

The endocrine and immune changes associated with estrogen deficiency after menopause have reshaped the role of NGF in bone. Estrogen plays a regulatory role in the NGF axis. Due to the estrogen deficiency, the sympathetic nervous system and the number of immune cells, especially T cells in the bone marrow, will increase and release a large number of inflammatory factors (56, 57). This indirectly affects the expression of NGF and its receptors. It was shown that TNF-α can be a strong inducer of more p75 NTR in osteoclast precursors and consequently augments the osteoclast differentiation that is induced by NGF (54, 55). Inflammatory substances like IL-1 and IL-6 may also trigger fibroblasts and glial cells to release NGF, which may increase its local concentration (58, 59). The elevation of NGF causes abnormal proliferation of sensory nerve endings in bone tissue and lowers the pain threshold, leading to chronic bone pain (14, 60).

Within the realm of PMOP, NGF facilitates adverse bone remodeling via two distinct mechanisms. On the one hand, NGF fosters osteoclastogenesis and leads to the induction of pain. On the other hand, NGF can mediate injury repair by promoting osteogenesis and neurovascular remodeling. Hence, it is possible to employ NGF as a therapeutic target; however, the strategy for intervention must be sensitive to the balance of the microenvironment. Clinically, Tanezumab, an anti-NGF neutralizing antibody, was used in clinical trials to alleviate pain in osteoarthritis and bone cancer, demonstrating pain relief. Nevertheless, some individuals with osteoarthritis show progressive joint damage (rapidly progressive osteoarthritis RPOA). Blocking NGF for a long time might cut back repair ability of joint and bone (15, 61). Following menopause, NGF levels and mechanisms are altered, acting as both a driving force and a feedback product of disrupted bone homeostasis. Therefore, it needs the research on its mechanisms for clarification.

## 4 Proposed gut microbiota–NGF–bone framework in postmenopausal bone homeostasis

### 4.1 Dysbiosis of gut microbiota and osteoporosis in postmenopause

Estrogen deficiency, which is believed to directly affect osteocyte function, also alters gut ecosystem. Following menopause or ovariectomy gut microbiota dysbiosis occurs, characterized by decreased diversity and increased growth of some potentially pro-inflammatory bacteria (62, 63). More importantly, gut barrier permeability is significantly increased after menopause: in conditions of low estrogen, tight junction protein expression in intestinal epithelial cells decreases, which limits the mucosal repair capacity and adds to the ‘leaky gut’ phenomenon (64). This allows a large number of bacterial products (such as LPS and peptidoglycan) to enter the bloodstream and the bone marrow microenvironment. This process activates the body’s immune system, particularly the gut-associated lymphoid tissue and T cells in the bone marrow. Research by Yu et al. has shown a significant increase in the number of Th17 cells and TNF+ T cells in the lamina propria of the gut in OVX mice, which migrate from the gut towards the bone marrow via S1P1 receptor signaling (65, 66). These pro-inflammatory T cells derived from the gut further produce cytokines such as IL-17 and TNF-α in the bone marrow, leading to a substantial expression of RANKL in osteoblasts and osteocytes, which directly stimulates osteoclast precursors, resulting in a rapid decrease in trabecular bone mass (67). Probiotic treatment has been shown to reduce bone resorption in OVX mice (68). These findings support an important contribution of microbiota-dependent immune activation to bone loss in OVX models; however, the magnitude and clinical relevance of this contribution in human PMOP remain uncertain. Along this axis, dysbiosis of the gut microbiota serves as the starting point, with impaired intestinal barrier functioning as the pathway, inflammatory T cells acting as effectors, and bone loss representing the ultimate phenotype.

### 4.2 Dysbiosis exacerbates bone homeostasis imbalance via NGF signaling

The systemic inflammation triggered by gut microbiota dysbiosis and autonomic nervous system imbalance provides an important pathological basis for the involvement of NGF in bone homeostasis regulation. Inflammatory factors significantly amplify the osteoclastogenic effects of NGF. Elevated levels of TNF-α and IL-17 post-menopause, in addition to promoting osteoclastogenesis directly through the RANKL pathway, can enhance the responsiveness of osteoclast precursors to NGF (69). These observations support a proposed feed-forward interaction between dysbiosis-associated inflammation and NGF-related signaling; however, this interaction has not been directly demonstrated as a complete causal loop in PMOP. At the same time, the activity of the sympathetic nervous system is also influenced by both gut microbiota and estrogen (70). Under physiological conditions, the sympathetic nervous system inhibits osteoblasts by releasing norepinephrine acting on β_2_ receptors, promoting osteoclastogenesis (71). After menopause, the inhibitory effect of estrogen on the sympathetic nervous system weakens, leading to increased sympathetic tone, which becomes a significant reason for enhanced bone resorption (72). Additionally, gut microbiota can also regulate autonomic nervous balance; probiotics can enhance vagal nerve activity and reduce sympathetic responses, whereas dysbiosis tends to lead to excessive sympathetic activation (36). Given the indispensable role of NGF in the survival of sympathetic and sensory neurons as well as axonal growth, the autonomic nervous adaptations induced by microbiota may involve the NGF pathway. Consequently, the intraosseous NGF and neural changes exhibit a complex relationship with osteoporosis. Mechanistically, these pathways may converge in the bone marrow microenvironment rather than functioning independently. A decrease in the availability of short-chain fatty acids and an increase in the displacement of microbial products may weaken intestinal barrier function and immune regulatory function, thereby promoting TNF-α and IL-17-related inflammation and enhancing RANKL-dependent osteoclastogenic pressure. Additionally, alterations in the balance between the vagus nerve and sympathetic nerve may affect osteoblast activity, sensory nerve and sympathetic nerve remodeling, as well as the local production of NGF or the tissue’s responsiveness to NGF. NGF signaling may serve as a context-dependent interface connecting these upstream signals with bone cells: TrkA-related signaling is associated with osteogenic differentiation and neurovascular coupling; whereas, during inflammatory states and changes in p75 NTR/TrkA expression, the overall effect may shift towards RANKL/OPG imbalance, osteoclast differentiation, and pain sensitization. Therefore, short-chain fatty acids, immune pathways, and autonomic nervous activity can be integrated into a proposed converging model, where inflammatory states and receptor expression backgrounds jointly determine whether NGF-related signals primarily manifest as reparative effects or catabolic effects. However, this interpretation integrates evidence from different experimental systems, and it cannot be concluded that NGF mediates the entire impact of gut microbiota dysbiosis on postmenopausal bone loss.

**Figure 2** presents an integrated, hypothesis-generating model in which menopause-associated dysbiosis may influence bone homeostasis through interacting metabolic, immune, autonomic, and NGF-related pathways. This interaction elevates inflammation levels, reduces vagal nerve activity, and enhances sympathetic tone, thereby altering the expression microenvironment of NGF and its receptors. This shift leads to a bone regulatory bias towards osteoclastogenesis and neural remodeling, ultimately exacerbating the imbalance of bone homeostasis. While the relevant mechanisms are still being explored, fragmented evidence is gradually being interconnected. Osteoporosis patients often present with chronic systemic inflammation, characterized by elevated levels of high-sensitivity C-reactive protein, and anti-inflammatory treatments (such as TNF inhibitors) can reduce osteoclastic activity and partially improve bone mass in rheumatoid patients (73). This underscores the potential value of blocking inflammation in amplifying the NGF/osteoclast axis. Furthermore, sympathetic nerve blockers or β-receptor antagonists have been shown to mitigate bone loss in animal models, indicating the feasibility of targeting the neuro-bone pathway for osteoporosis intervention (74).

**Figure 2:**
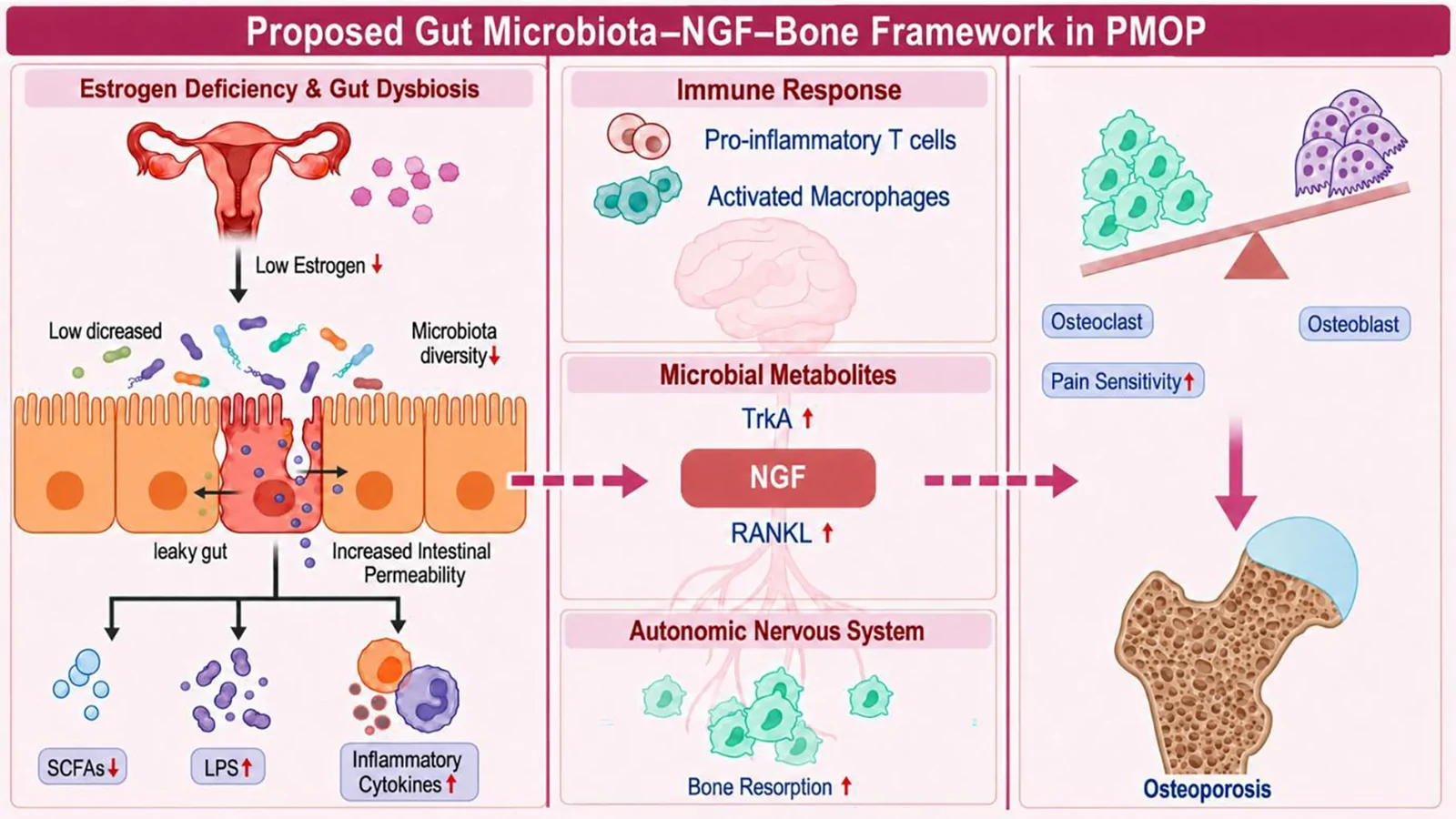
Proposed gut microbiota–NGF–bone framework relevant to postmenopausal osteoporosis. Estrogen deficiency leads to gut dysbiosis, reduced microbial diversity, and increased intestinal permeability, resulting in decreased short-chain fatty acids (SCFAs), elevated lipopolysaccharides (LPS), and higher levels of inflammatory cytokines. These changes activate pro-inflammatory T cells and macrophages, and influence bone metabolism via microbial metabolites, the autonomic nervous system, and NGF-related signaling pathways. The diagram integrates independently supported microbiota–immune–bone and NGF/receptor–nerve–bone pathways. The proposed links between microbiota-induced changes in NGF signaling and skeletal outcomes remain unvalidated and should be interpreted as hypothesis-generating rather than causal.

### 4.3 Indirect evidence from animal and clinical studies relevant to the proposed framework

Animal studies have provided experimental evidence for individual components relevant to the proposed framework, but no study has directly validated the complete gut microbiota–NGF–bone causal chain. Knockout of the NGF receptor p75 NTR leads to exacerbated intestinal inflammation and worsened bone destruction in mice, suggesting that under normal conditions, NGF/p75 NTR signaling helps to limit inflammation and protect the skeleton (40, 75). Zhao et al. (52) conducted conditional knockout of p75 NTR in an animal model of osteoarthritis, which resulted in a more intense inflammatory response and impaired cartilage and bone remodeling. This indicates that NGF/p75 NTR signaling plays an anti-inflammatory and repair-promoting role in joint homeostasis. It suggests that moderate NGF signaling, through pathways such as p75 NTR, suppresses excessive inflammation, thereby maintaining the balance of the bone/cartilage environment. If this is extended to the gut-bone axis, it can be inferred that the maintenance of gut microbiota health (absence of pathogenic inflammation) is beneficial for maintaining bone homeostasis through NGF signaling.

Recent clinical studies have compared the gut microbiota of postmenopausal women undergoing hormone replacement therapy (HRT) with those not receiving HRT. HRT women have been associated with higher α-diversity of fecal microbiota with enrichment of butyrate-producing bacteria such as Faecalibacterium and Bacteroides; while the untreated group is enriched in potential pathogens such as Escherichia coli-Shigella (76). HRT therapy results in low levels of the markers of bone metabolism, type I pro-collagen amino-terminal propeptide (P1NP) and type I collagen C-terminal peptide (CTX-1). The lowered levels of these biomarkers indicate the slower rate of bone turnover and rate of bone loss. This leads to the speculation that bacterial communities in the intestine may influence bone homeostasis through changes in systemic inflammatory and hormonal metabolic environments. While these clinical observations do not measure NGF directly, it is reasonable to deduce that the physiological balance determined by a healthy microbiota in conjunction with the low-inflammation, high-estrogen milieu created by HRT, may favor NGF signaling towards the osteogenic/anti-inflammatory side rather than the osteoclastic/algogenic side.

### 4.4 Current evidence and key limitations

Existing literature supports several components of the proposed “gut microbiota–NGF–bone” theoretical framework; however, there are significant differences in the strength of evidence for different components and their correlation with postmenopausal osteoporosis. Direct experiments have confirmed that, under specific neuroimmune conditions, gut microbiota can regulate NGF; gut microbiota can influence bone metabolism through immune mechanisms; and NGF receptors are also involved in skeletal biological processes. Nevertheless, these results stem from independent experimental systems. Currently, no cited studies have simultaneously manipulated gut microbiota and NGF signaling in estrogen-deficient models, nor have they further confirmed the bone phenotype mediated by NGF. Therefore, **Table 1** distinguishes between the components that have received experimental support and those that are supported by indirect or correlational evidence, summarizing the main limitations of each type of evidence.

**Table 1:** Studies relevant to gut microbiota-mediated regulation of NGF and related signaling.

Study	Model	Intervention/exposure	NGF/receptor change	Bone phenotype	Proposed pathway
Abdullah et al., 2025 (22)	Germ-free and conventionally colonized mice; early-life colonization paradigm	Microbiota absence or colonization during early life	Colonization increased mast-cell-derived NGF; downstream TrkA-dependent nociceptor sensitivity was restored	Not assessed	Microbiota colonization → mast-cell maturation/NGF release → TrkA activation → TRPV1 trafficking and nociceptor sensitization
Caroleo et al., 2001 (25)	Cultured human monocytes/macrophages	Lipopolysaccharide (LPS) exposure	NGF and NGF-receptor expression increased after LPS stimulation	Not assessed	Microbial product (LPS) → monocyte/macrophage activation → increased NGF/NGF-receptor expression
Song et al., 2025 (34)	Murine irritable bowel syndrome model	Oral dual-Lactobacillus combination	Brain NGF and BDNF levels increased; bone-localized NGF was not measured	Not assessed	Probiotic-induced microbiota/immune modulation → vagal and central neurotrophic responses
Barichello et al., 2015 (18)	Rat experimental pneumococcal meningitis model	Sodium butyrate supplementation	NGF was not measured; hippocampal BDNF and GDNF expression was restored	Not assessed	SCFA exposure → neurotrophic-factor-related regulation; relevance to NGF remains indirect
Nohr et al., 2013 (20)	Enteroendocrine cells, enteric neurons, and intestinal leukocytes	SCFA receptor-expression/signaling analysis	NGF and its receptors were not measured	Not assessed	SCFAs → FFAR3 in enteric neurons and FFAR2 in intestinal leukocytes; a potential upstream route rather than direct NGF evidence
Saikachain et al., 2023 (21)	Oxidative-stress-injured SH-SY5Y neuronal cells	Short-chain fatty acids	NGF was not measured	Not assessed	SCFAs → GPR43-dependent neuroprotection; indirect support for metabolite-neural signaling
Qiao et al., 2025 (38)	Human genetic Mendelian-randomization and mediation analyses	Genetically predicted gut-microbiota traits; no administered intervention	NGF and its receptors were not measured	Associations with bone mineral density; autonomic indices partly mediated selected associations	Gut-microbiota traits → autonomic nervous function → bone density; NGF mediation remains untested

The table deliberately distinguishes direct NGF measurements from studies that support only an upstream metabolite, immune, or autonomic mechanism. None of the listed studies simultaneously assessed microbiota manipulation, NGF-dependent mediation, and a skeletal outcome in an estrogen-deficient model. BDNF, brain-derived neurotrophic factor; FFAR, free fatty acid receptor; GDNF, glial cell line-derived neurotrophic factor; NGF, nerve growth factor; SCFA, short-chain fatty acid; TRPV1, transient receptor potential vanilloid 1.

In summary, current evidence most strongly supports two parallel but not yet fully interconnected pathways: the “gut microbiota–immune–bone” pathway and the “NGF/receptor–nerve–bone” pathway. At this stage, there is no evidence to suggest that gut microbiota-induced changes in NGF are a necessary or sufficient condition for postmenopausal bone remodeling. Therefore, the complete “gut microbiota–NGF–bone” axis should be articulated as a conceptual framework awaiting validation. Future research should combine manipulation of gut microbiota with NGF or its receptor blockade, cell-specific genetic interventions, rescue experiments, and concurrent skeletal phenotype analyses.

## 5 Focused therapy of gut microbiota for PMOP

### 5.1 The role of probiotics and prebiotics in improving bone homeostasis

Research activities regarding interventions that target gut microbiota are among the most active in relation to the gut-bone axis. Several studies have shown that probiotics or prebiotics supplementation can improve bone health (77). Probiotics can support the establishment of beneficial gut bacteria and enhance gut bacteria’s metabolic activity, leading to better synthesis of short-chain fatty acids and reinforcement of gut barrier function. As a result, there is a reduction in systemic inflammation and better calcium absorption (78). Tyagi et al. provided normal mice with Lactobacillus rhamnosus GG (LGG) for a certain period of time and reported a significant increase in trabecular bone volume, which was attributed to the enhancement of osteoblasts and not by inhibition of osteoclasts (79). Researchers noted the ability of LGG to modify the profile of intestinal metabolites, enhance the spectrum of butyrate-producing bacteria and induce the Treg cells’ expansion in the gut and bone marrow (80). In the bone marrow, Treg cells release TGF-β that interacts with CD8+ T cells. The result is the production of Wnt10b from CD8+ T cells, which activates osteogenesis. Wnt10b is an important ligand that promotes differentiation and activation of osteoblasts, which enhances bone formation. Wnt/β-catenin pathway is stimulated in bone marrow stromal cells and osteoblasts. For a while now, research has indicated that probiotics are capable of improving bone health.

Prebiotics, such as oligofructose and inulin, serve as “food” for beneficial bacteria and can promote the proliferation of intestinal acid-producing bacteria. Mouse experiments have shown that prebiotics can increase the concentration of short-chain fatty acids (SCFAs), directly inhibit the formation of osteoclasts, and promote the activity of osteoblasts (81, 82). SCFAs, particularly propionate and butyrate, can alter the epigenetic status of genes associated with NGF, thereby indirectly regulating the production and release of NGF. Moreover, the immune effects of probiotics and prebiotics are not limited to Treg/Th17 balance. They may also regulate macrophage polarization by decreasing pro-inflammatory M1 macrophages and their related cytokines, such as TNF-α and IL-6, while increasing anti-inflammatory M2 macrophages, which produce TGF-β and IL-10. This M1-to-M2 shift helps reduce inflammatory bone resorption and supports osteoblast-mediated bone formation.

Existing evidence suggests that probiotics and prebiotics may primarily improve bone-related outcomes by promoting the production of SCFAs, protecting the intestinal barrier, and regulating calcium metabolism and immune responses. Whether NGF or autonomic nervous regulation mediates these skeletal effects remains a hypothesis. Increasing dietary fiber intake may be beneficial for overall health; however, its efficacy in preventing PMOP and whether it relies on NGF still require well-designed clinical trials for confirmation.

### 5.2 Effects of antibiotic intervention on gut microbiota and bone metabolism

Unlike probiotic supplementation, broad-spectrum antibiotics can alter the gut microbiota, even leading to a transient clearance of these microorganisms, which in turn affects bone metabolism. In postmenopausal models, short-term antibiotic intervention can prevent bone loss to some extent. Li et al. administered an antibiotic cocktail to OVX mice to disrupt their gut microbiota, which significantly reduced the number of Th17 cells and osteoclasts in the bone marrow of OVX mice, consequently decreasing the extent of bone loss compared to untreated OVX controls (83). This confirms the role of gut microbiota in PMOP and indicates that regulating the microbiota (including using antibiotics for suppression) has therapeutic potential under specific circumstances. However, if one manages to get rid of the gut microbiota completely, it will be challenging to keep that up for the long term, which may further harm overall health in various aspects (immune defense, metabolism, etc.). One possibility is to inhibit harmful microbes or their metabolic products, but this may be easier said than done (84).

In addition, future interventions may focus on reducing pro-inflammatory macrophage activation. For example, inhibiting the M1 macrophage phenotype and lowering the production of TNF-α and IL-6 may help reduce bone marrow inflammation and osteoclast activation. Conversely, promoting M2 macrophage polarization and enhancing TGF-β and IL-10 production may support an anti-inflammatory microenvironment favorable for bone maintenance. Blocking gut permeability factors or T cell migration pathways could be considered to reduce bone marrow inflammation. It may be possible to develop a narrow-spectrum antibiotic or inhibitor that targets pro-inflammatory gut bacteria in the future, or otherwise inhibits the binding of key metabolic products from these bacteria (such as lipopolysaccharide, LPS), to specific host receptors (e.g. TLR4 antagonists). However, these agents should elicit bone-protective effects similar to those of antibiotic intervention, but without disturbing the whole microbiota.

### 5.3 The regulatory role of hormone replacement therapy on the gut-bone axis

Hormone replacement therapy (HRT) after menopause is an effective way of preventing osteoporosis. HRT acts directly on bone and can also influence the gut microbiota to exert some effects. Research by Xiong et al. found that postmenopausal women who used HRT displayed the high abundance of beneficial bacterial genera from the phylum Firmicutes in their intestines, while the abundance of potentially pathogenetic genera, namely from the phylum Proteobacteria, was reduced (76). At the same time, the P1NP and CTX markers of bone turnover decreased in these women. These findings suggest that HRT may regulate the gut-bone axis through both microbial and immune mechanisms. By improving gut microbial composition and reducing gut-derived inflammatory stimulation, HRT may help suppress pro-inflammatory immune responses, including Th17 cell activation and IL-17 production.

Certain genera enriched in the HRT group, such as bacteria from the Lachnospiraceae family, can produce phytoestrogen metabolites, such as isoflavones generated by the genus Adlercreutzia, which further exert estrogen-like effects in the body, beneficial for bone protection (85). HRT also positively affects the intestinal mucosal barrier: estrogens promote the proliferation of intestinal epithelial cells and mucus secretion, repair tight junctions in the gut, thereby reducing the translocation of bacteria and endotoxins (86, 87). This suggests that HRT can regulate the quantity of beneficial and pathogenic bacterial genera, balancing the inhibition of osteoclasts and reducing bone resorption, while also maintaining gut integrity to decrease inflammatory triggers and lower osteoclast stimulation.

Although there is currently no direct evidence proving that Hormone Replacement Therapy (HRT) affects NGF, considering the role of estrogen in the nervous system (such as enhancing the expression of central BDNF and NGF), it is hypothesized that HRT as a whole may contribute to maintaining the balance of the gut microbiota–NGF–bone axis, allowing mediators like NGF to remain at levels conducive to bone formation and inflammation suppression. In clinical practice, for women who cannot use hormone replacement therapy (HRT) (due to potential increased risks of breast cancer, thrombosis, etc.), it is worth exploring whether adjusting the gut microbiota could partially substitute for the bone protective effects of HRT. Given the significant heterogeneity in experimental models, types of interventions, and outcome indicators across different studies, it is necessary to conduct a structured comparison to evaluate the translational value of these strategies. **Table 2** summarizes the aforementioned gut microbiota-targeted interventions, including the research models, main findings, effects on gut microbiota, skeletal outcomes, and reported associations with NGF. It is noteworthy that the existing evidence predominantly comes from preclinical studies, and the cited skeletal intervention studies have not directly demonstrated that NGF mediates the impact of microbiota modulation on bone homeostasis.

**Table 2:** Gut microbiota-targeted intervention strategies relevant to bone homeostasis.

Strategy	Model/population	Main finding	Gut microbiota effect	Skeletal outcome	NGF association	Reference
Probiotic mixture	Ovariectomized mice	A defined Lactobacillus probiotic mixture attenuated estrogen-deficiency-associated inflammatory responses	Microbiota-targeted probiotic exposure; the study focused primarily on immune effects rather than comprehensive community profiling	Protected against cortical bone loss	Not measured; no demonstrated NGF mediation	Ohlsson et al., 2014 (68)
Bifidobacterium animalis subsp. lactis A6	Preclinical mouse model of age-related bone and muscle loss	B. lactis A6 improved bone-muscle homeostasis and increased butyrate-related metabolic capacity	Remodeled microbial composition and enhanced butyrate production	Ameliorated bone loss and improved bone-related indices	Not measured; a butyrate-NGF link is only hypothetical	Chen et al., 2025 (77)
Butyrate/probiotic-associated metabolite	Mice; Treg- and CD8+ T-cell-dependent bone-formation experiments	Butyrate expanded regulatory T cells and induced CD8+ T-cell Wnt10b production	Uses a microbial metabolite; microbiota composition was not the primary endpoint	Stimulated osteoblast activity and bone formation	Not measured; effect was linked to the Treg-CD8+ T-cell-Wnt10b pathway	Tyagi et al., 2018 (79)
FOS/GOS prebiotics	High-fat-diet-fed mice	FOS/GOS improved intestinal permeability and reduced systemic inflammation	Increased microbial diversity and SCFA concentrations and reversed dysbiosis	Attenuated bone loss; improved osteoblast/osteoclast differentiation balance	Not measured; any SCFA-NGF connection remains unverified	Zhang et al., 2021 (81)
Metabolically augmented synbiotic microspheres	Preclinical bone/joint disease model	Self-replenishable synbiotic delivery remodeled gut-bone homeostasis	Combined probiotic/prebiotic metabolic augmentation and microbiota remodeling	Improved bone-related outcomes in the reported preclinical model	Not measured	Chen et al., 2025 (82)
Microbiota depletion and probiotic rescue	Sex-steroid-deficient germ-free, antibiotic-treated, and conventionally raised mice	Microbiota depletion prevented key immune changes, whereas LGG or VSL#3 reduced gut permeability and inflammation	Direct depletion or probiotic modification of the intestinal microbiota	Prevented or attenuated sex-steroid-deficiency-associated bone loss	Not measured; supports a microbiota-immune-bone pathway independent of demonstrated NGF mediation	Li et al., 2016 (83)
Tryptophan-producing bacteria	Preclinical osteoporosis and intestinal-dysfunction model	Engineered/selected bacteria restored microbial tryptophan-derived metabolites and improved intestinal function	Increased microbiota-derived tryptophan metabolites and remodeled gut metabolic activity	Mitigated osteoporosis-related skeletal deterioration	Not measured	Tian et al., 2025 (84)
Menopausal hormone therapy	Postmenopausal women receiving versus not receiving hormone therapy; observational comparison	Hormone therapy was associated with microbial differences and lower bone-turnover markers	Higher alpha-diversity and enrichment of selected butyrate-producing taxa; fewer potential pathogens	Lower P1NP and CTX; fracture or BMD benefit was not established by this observational study	Not measured; microbial mediation and NGF involvement cannot be inferred	Xiong et al., 2025 (76)

Most interventions were evaluated in preclinical models, and NGF was not measured in the cited skeletal intervention studies. Accordingly, the NGF association column should not be interpreted as evidence that NGF mediates the skeletal effects. BMD, bone mineral density; CTX, C-terminal telopeptide; FOS, fructooligosaccharides; GOS, galactooligosaccharides; LGG, Lactobacillus rhamnosus GG; NGF, nerve growth factor; P1NP, procollagen type I N-terminal propeptide; SCFA, short-chain fatty acid; Treg, regulatory T cell.

As summarized in **Figure 3**, probiotics/prebiotics, dietary modification, antibiotics, and hormone replacement therapy (HRT) have been investigated as potential modulators of microbiota-related pathways relevant to bone homeostasis in PMOP. These interventions may act by modulating the composition of gut microbiota, promoting the production of short-chain fatty acids, and repairing intestinal barrier function, or by inhibiting pro-inflammatory pathways, regulating immune cell activity, and reducing osteoclastic stimulation. Ultimately, they work together on the bone metabolism process to promote bone formation and inhibit bone resorption, providing strategies for the clinical prevention and treatment of PMOP.

**Figure 3:**
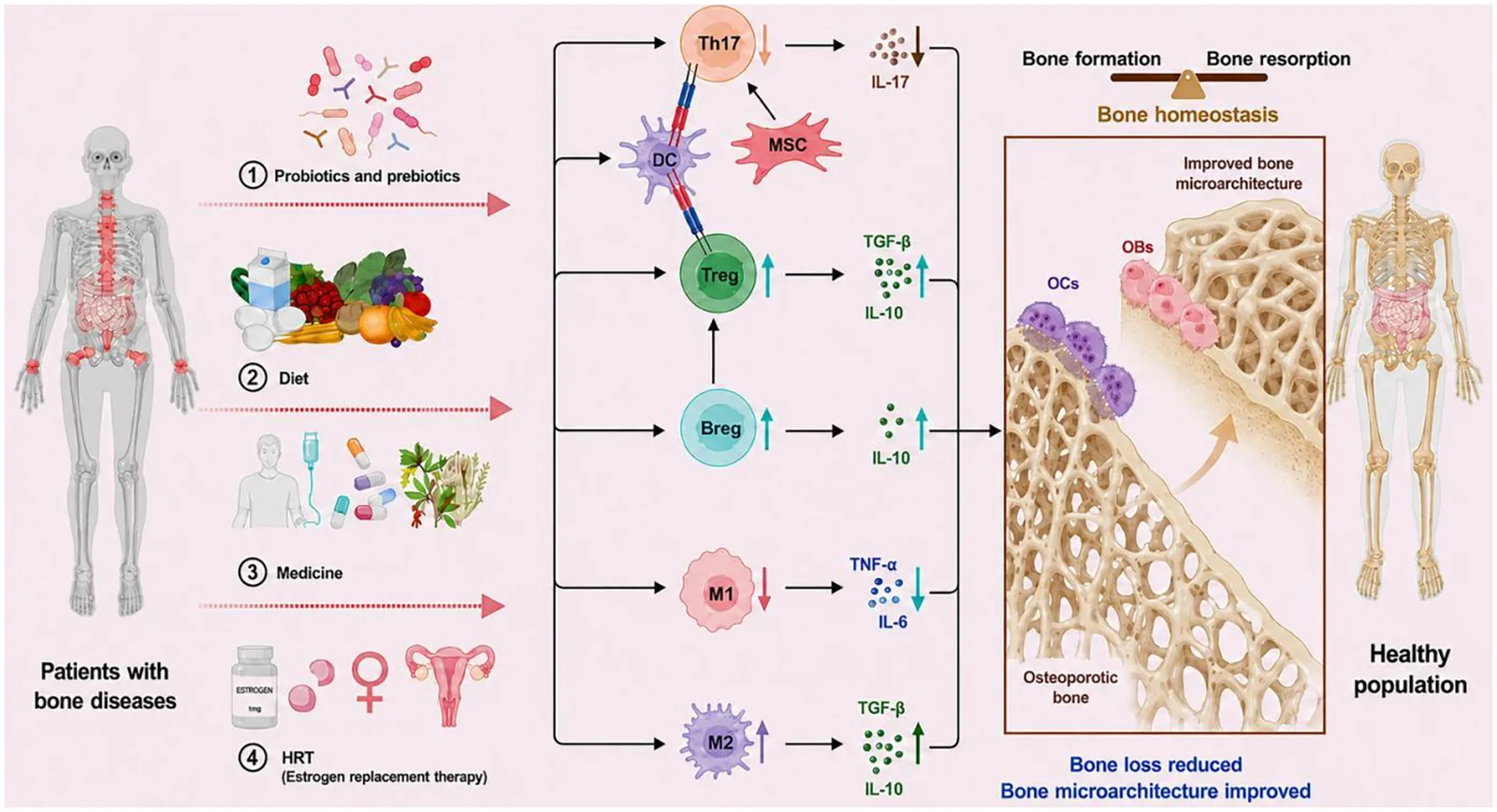
Gut microbiota-targeted interventions and immune regulatory pathways in postmenopausal osteoporosis. The figure illustrates the targeted and systemic intervention strategies related to osteoporosis, including probiotics/prebiotics, dietary adjustments, pharmacological treatments, and hormone replacement therapy. These interventions may modulate the gut microbiota and the activity of immune cells such as Treg, Breg, Th17, and M1/M2 macrophages, thereby altering the signaling of IL-10, TGF-β, TNF-α, IL-6, and IL-17. The resulting immune changes may reduce osteoclastogenic pressure, support osteoblast activity, decrease bone loss, and improve trabecular microarchitecture. This figure represents a proposed integrative model and does not demonstrate that NGF mediates these intervention effects.

## 6 Conclusion

PMOP is a bone metabolic disease caused by estrogen deficiency. Its occurrence and development involve not only classic endocrine-immunological regulation but may also be influenced by the interplay of gut microbiota, neural regulatory networks, and the bone microenvironment. Existing studies indicate that gut microbiota and their metabolites can affect bone remodeling by regulating intestinal barrier function, immune inflammatory responses, and autonomic nervous activity. NGF and its receptors TrkA and p75 NTR are involved in osteoblast differentiation, sensory nerve innervation, bone repair, osteoclastogenesis, and pain regulation. Based on this independent evidence, the gut microbiota-NGF-bone axis can serve as a conceptual framework linking microbiology, immunity, neuroendocrinology, and the bone metabolic system, providing insights for understanding the complex pathological mechanisms of PMOP and exploring new intervention targets.

Currently, there is a lack of direct evidence to fully validate the causal chain of “gut microbiota changes - NGF signal variations - bone remodeling abnormalities.” Existing studies primarily support the gut microbiota-immune-bone pathway and the NGF/receptor-neural-bone pathway separately, but they do not demonstrate that NGF is a necessary or sufficient mediator for the influence of gut microbiota on postmenopausal bone metabolism. Moreover, the biological effects of NGF are significantly dependent on the microenvironment, and its effects may vary with inflammatory status, estrogen levels, dosage, target cell types, receptor composition, and disease stages. The existing evidence mainly comes from animal experiments, *in vitro* studies, and observational analyses, which face issues such as heterogeneous research models, limited clinical sample sizes, insufficient integration of multi-omics data, and inadequate control of confounding factors like diet, medication, and chronic inflammation. Therefore, at this stage, it is inappropriate to characterize the gut microbiota-NGF-bone axis as an established pathological mechanism or therapeutic pathway, and interventions targeting this axis, such as probiotics, prebiotics, neuroregulation, and NGF-related therapies, require further validation.

Future research should prioritize direct mechanism verification. In models of ovariectomy or estrogen deficiency, interventions using germ-free animals, fecal microbiota transplantation, clearly defined microbial consortia, or microbial metabolites can be combined with cell or tissue-specific knockouts, blocking, and activation of NGF, TrkA, and p75 NTR. The necessity and sufficiency of NGF in microbiota-related bone phenotypes can be assessed through blocking and rescue experiments. Simultaneously, within the same longitudinal study framework, the composition and function of the microbiota, microbial metabolites, intestinal barrier permeability, immune cell migration, autonomic nervous activity, circulating and local bone NGF signaling, bone turnover markers, bone microstructure, and mechanical properties should be synchronously measured to clarify the temporal sequence and causal relationships of each component. At the clinical level, prospective cohort studies and randomized controlled trials controlling for major confounding factors are also needed, combined with pre-defined mediation effect analyses to distinguish between the direct bone effects of treatment and microbiota- and NGF-dependent actions, as well as to systematically evaluate fracture prevention, treatment durability, neuro-immune safety, and long-term impacts on microbial ecology. Through these studies, the relevant hypotheses regarding the gut microbiota–NGF–bone axis can be further confirmed, refined, or refuted, providing a more reliable theoretical basis for the precise prevention and individualized treatment of PMOP.
